# Regulation of SKP2 protein stability by heat shock protein 90 chaperone machinery

**DOI:** 10.1038/s41392-021-00624-1

**Published:** 2021-07-30

**Authors:** Lili Cai, Lihui Li, Xihui Chen, Lijun Jia

**Affiliations:** Cancer Institute, Longhua Hospital, Shanghai University of Traditional Chinese Medicine, Shanghai, China

**Keywords:** Oncogenes, Drug regulation

**Dear Editor**,

S phase kinase-associated protein 2 (SKP2), a substrate recognition component of the SCF^SKP2^ ubiquitin ligase complex, plays an oncogenic role in tumorigenesis by targeting a variety of tumor suppressors (e.g., p21, p27, and p130) for ubiquitination and subsequent degradation.^1^ As a well-characterized oncoprotein, the aberrant expression and dysregulation of SKP2 are frequently observed in different human cancers. Given its critical role in governing tumorigenesis and progression, SKP2 has emerged as a potential pharmacological target for anticancer therapy.^1^

Heat shock protein 90 (HSP90) is an evolutionarily conserved molecular chaperone that participates in stabilizing and activating >200 proteins termed HSP90 client proteins.^2^ In addition, HSP90 is highly expressed in various tumors compared with normal tissues to maintain the stabilization of numerous client oncoproteins. Therefore, several small-molecule inhibitors (e.g., 17-AAG and STA-9090) that target HSP90 to inhibit its ATPase activity have been developed as potential anticancer drugs and undergo clinical trials in various human cancers.^2^ Given that SKP2 is sophisticatedly regulated in human tumors, whether it can be directly regulated by HSP90 remains poorly understood.

In order to examine whether SKP2 is regulated by HSP90 chaperone machinery, two HSP90 small-molecule inhibitors, 17-AAG and STA-9090, were applied to treat several human cancer cell lines (A549, H1299, MDA-MB-231, and SW480). It was observed that treatment with 17-AAG or STA-9090 downregulated SKP2 protein expression, and subsequently led to the accumulation of p27, the most classical substrate of SKP2 (Fig. 1a, b). AKT, a known client protein for HSP90, was used as a positive control of HSP90 inhibition (Fig. 1a, b). As for the mechanism investigation, we found that treatment of 17-AAG or STA-9090 had little effect on *SKP2* mRNA levels (Supplementary Fig. S1). To further ascertain how HSP90 inhibitors downregulate SKP2 expression, we examined the effect of 17-AAG on the stability of SKP2. To this end, we applied cycloheximide to block new protein synthesis and detected SKP2 degradation with or without 17-AAG treatment. As shown in Fig. 1c, 17-AAG treatment shortened the half-life of SKP2 (Fig. 1c). In order to verify the HSP90–SKP2–p27 axis, we further examined whether SKP2 rescue could abrogate 17-AAG/STA-9090-induced upregulation of p27, and found that SKP2 overexpression partially rescued the p27 accumulation upon treatment of 17-AAG or STA-9090 (Supplementary Fig. S2). Collectively, these data demonstrate that HSP90 inhibitors promote SKP2 degradation and subsequent p27 accumulation.

**Fig. 1:**
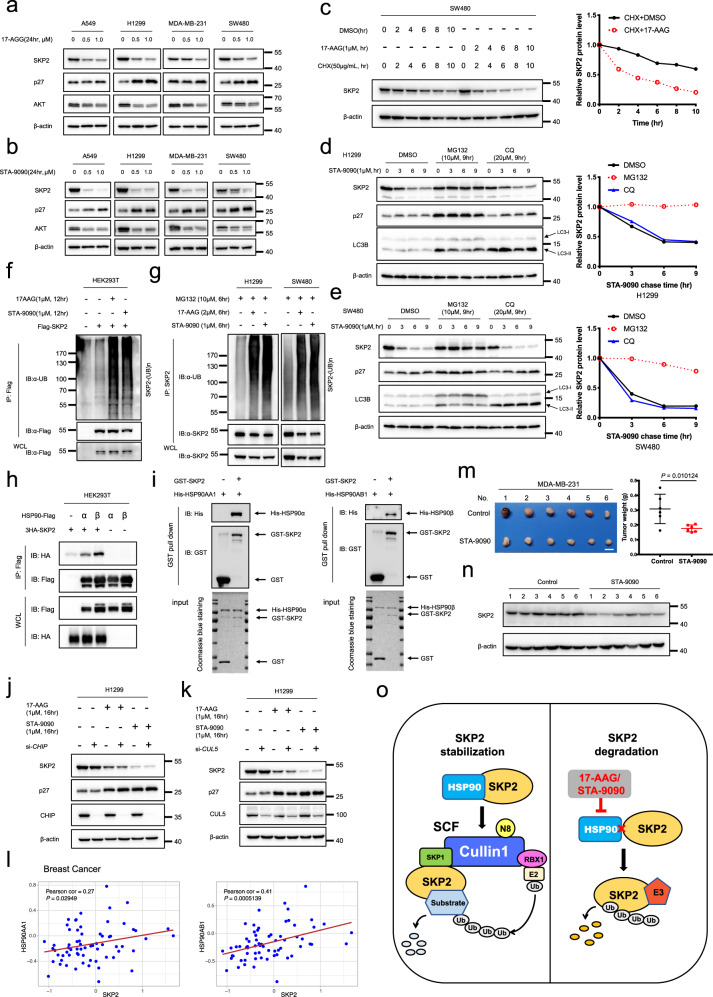
HSP90 inhibitors induce SKP2 ubiquitination and proteasomal degradation. **a**, **b** 17-AAG and STA-9090 treatment downregulated SKP2 in a dose-dependent manner in various cancer cells. **c** 17-AAG treatment promoted the degradation of SKP2 in SW480 cells. The band density of SKP2 was quantified by densitometric analysis, using ImageJ software (right panel). **d**, **e** STA-9090-induced SKP2 degradation was blocked by MG132 treatment, but not by chloroquine treatment. p27 was detected as a positive control of MG132 treatment, while LC3B was used as a positive control of CQ treatment. The band density of SKP2 was quantified by densitometric analysis, using ImageJ software (right panel). **f** 17-AAG and STA-9090 induced the poly-ubiquitination of SKP2 in HEK293T cells transfected with Flag-tagged SKP2. **g** 17-AAG and STA-9090 induced the poly-ubiquitination of SKP2 in H1299 and SW480 cells. **h** SKP2 interacted with both HSP90α and HSP90β. **i** SKP2 directly bound to HSP90α and HSP90β. **j** CHIP knockdown using siRNA did not compromise HSP90 inhibitor-induced SKP2 degradation. **k** CUL5 knockdown using siRNA did not compromise HSP90 inhibitor-induced SKP2 degradation. **l** Pearson’s correlation analysis revealed the positive correlation between HSP90 and SKP2 in breast cancer. **m** Mice were sacrificed and tumor tissues were harvested and photographed. Scale bar = 1 cm. The tumor weight was measured with an electronic scale on the sacrificed day. Student’s *t* test was used for the significance analysis. **n** Proteins were extracted from tumor tissues and subjected to immunoblotting analysis against SKP2 with β-actin as a loading control. **o** A model of the possible mechanism underlying the regulation of SKP2 by HSP90 chaperone machinery.

We further determined whether HSP90 inhibitor-induced degradation of SKP2 was regulated through ubiquitin–proteasome pathway or lysosome degradation pathway. As the result shown, STA-9090-induced protein degradation of SKP2 was obviously blocked by MG132 (a classical proteasome inhibitor), but neither by CQ (chloroquine) nor bafilomycin A1 (both are classical lysosomal inhibitors; Fig. 1d, e and Supplementary Fig. S3), indicating that STA-9090 treatment induces SKP2 degradation in a proteasome-dependent manner. Moreover, we examined whether SKP2 underwent ubiquitination upon treatment with HSP90 inhibitors, and found that the levels of SKP2 poly-ubiquitination were significantly enhanced upon treatment with either 17-AAG or STA-9090 (Fig. 1f, g and Supplementary Fig. S4), suggesting that HSP90 inhibitors induce SKP2 ubiquitination and subsequently proteasome-mediated degradation. Meanwhile, we tested whether HSP90 isoforms bind to SKP2. As shown in Fig. 1h, SKP2 interacted with both HSP90α and HSP90β under ectopic overexpression condition (Fig. 1h). Furthermore, in vitro purified GST-SKP2 effectively pulled down both His-HSP90α and His-HSP90β (Fig. 1i), further indicating that HSP90 directly binds to SKP2 and regulates its stability.

The C terminus of Hsc70-interacting protein (CHIP, also known as STUB1) is one of the major E3 ubiquitin ligases involved in the HSP90 chaperone system.^3^ We thus tested whether CHIP serves as an E3 ligase mediating SKP2 degradation upon the treatment of HSP90 inhibitors, and found that CHIP knockdown did not rescue 17-AAG or STA-9090-induced SKP2 degradation (Fig. 1j and Supplementary Fig. S5a). Likewise, ectopic overexpression of CHIP had no effect on the SKP2 expression (Supplementary Fig. S5b). Thus, these results suggest that HSP90 inhibitor-induced SKP2 degradation is independent of CHIP. Besides CHIP, Cullin5 (CUL5) E3 ligase also mediates ubiquitin-dependent degradation of HSP90 client proteins.^3^ However, CUL5 knockdown also had no effect on HSP90 inhibitor-induced SKP2 degradation (Fig. 1k and Supplementary Fig. S5c). Furthermore, we examined whether other Cullin-Ring E3 ligases (CRLs) contribute to HSP90 inhibitor-induced SKP2 degradation. To this end, we treated cells with MLN4924, the specific NEDD8-activating enzyme inhibitor that inhibits Cullin neddylation and induces CRLs inactivation, and found that MLN4924 treatment did not compromise STA-9090-induced SKP2 degradation (Supplementary Fig. S5d).

Since CDH1 is a known E3 ligase to mediate SKP2 destruction,^4^ thus we tested whether CDH1 is responsible for HSP90 inhibitor-induced SKP2 degradation. It was observed that knockdown of CDH1 induced SKP2 accumulation (Supplementary Fig. S6, lane 1 vs lane 2), but only modestly compromised HSP90 inhibitor-induced SKP2 degradation (Supplementary Fig. S6), indicating that HSP90 inhibitor-induced SKP2 degradation was regulated by an unknown E3 ligase other than CDH1. In order to identify the putative E3 ligase involved in HSP90 inhibitor-induced SKP2 degradation, we performed proteomic analysis of SKP2-interacting proteins after STA-9090 and MG132 treatment. The putative E3 candidates were listed in Supplementary Fig. S7a. Unfortunately, knockdown of any one of these E3 candidates (RNF219, DTX3L, UBR5, RING1, RNF2, and AMFR) did not rescue HSP90 inhibitor-induced SKP2 degradation (Supplementary Fig. S7b–g), suggesting that none of these candidates are involved in the downregulation of SKP2 upon HSP90 inhibition. Taken together, these findings indicate that additional to-be-identified E3 ligase is responsible for the SKP2 degradation upon HSP90 inhibitors treatment.

Given that HSP90 and SKP2 are frequently overexpressed in tumors, we further detected the correlation between HSP90 and SKP2 protein expression in tumor samples. The CPTAC proteomics data of Breast Cancer^5^ (https://proteomic.datacommons.cancer.gov/pdc/) were examined, and Pearson’s correlation analysis validated the positive correlation between HSP90 and SKP2 in breast cancer (Fig. 1l), indicating that the overexpression of HSP90 contributes to stabilizing SKP2 in tumors.

Furthermore, we assessed the therapeutic potential of STA-9090 in MDA-MB-231 subcutaneous transplantation tumor model. As shown, STA-9090 treatment significantly inhibited tumor growth, as analyzed by tumor weight (*P* < 0.05, Fig. 1m), and downregulated SKP2 expression in xenografted nude mice (Fig. 1n). Unexpectedly, p27 was not accumulated in mice tumor samples of STA-9090 treatment group (data not shown), which may due to the complicated role of HSP90 inhibitors and sophisticated regulation of p27 in vivo.

In conclusion, the present study demonstrates that SKP2 is regulated by HSP90 chaperone machinery and undergoes poly-ubiquitination targeted proteasomal degradation upon HSP90 inhibition (Fig. 1o). Given that the overexpression of both HSP90 and SKP2 have been observed in a number of cancers, overexpressed HSP90 assists the folding and stabilization of SKP2 to promote cancer progression through mediating the degradation of tumor-suppressive substrates (Fig. 1o).

## Supplementary information

Supplementary material file

## References

[1] Wang Z, Liu P, Inuzuka H, Wei W (2014). Roles of F-box proteins in cancer. Nat. Rev. Cancer.

[2] Trepel J, Mollapour M, Giaccone G, Neckers L (2010). Targeting the dynamic HSP90 complex in cancer. Nat. Rev. Cancer.

[3] Schopf FH, Biebl MM, Buchner J (2017). The HSP90 chaperone machinery. Nat. Rev. Mol. Cell Biol..

[4] Gao D (2009). Phosphorylation by Akt1 promotes cytoplasmic localization of Skp2 and impairs APCCdh1-mediated Skp2 destruction. Nat. Cell Biol..

[5] Krug K (2020). Proteogenomic landscape of breast cancer tumorigenesis and targeted therapy. Cell.

