# Supplementary material for: Regulation of SKP2 protein stability by heat shock protein 90 chaperone machinery
Source: Signal Transduct Target Ther. 2021 Jul 30;6:276. doi: 10.1038/s41392-021-00624-1 (PMC8322090; doi:10.1038/s41392-021-00624-1)
Supplement: Supplementary file 1 — Supplementary material file [file 41392_2021_624_MOESM1_ESM.docx]

Supplementary Materials for

Regulation of SKP2 protein stability by heat shock protein 90 chaperone machinery

Lili Cai^1#^, Lihui Li^1#^, Xihui Chen^1^, Lijun Jia^1^

^1^Cancer Institute, Longhua Hospital, Shanghai University of Traditional Chinese Medicine, Shanghai 200032, China.

# These authors contributed equally to this work.

Correspondence to: Lijun Jia (ljjia@shutcm.edu.cn)

**This PDF file includes:**

Materials and Methods

Figures. S1 to S7

Materials and Methods

**Reagents and antibodies**

17-AAG and STA-9090 were purchased from Selleck. Cycloheximide was purchased from Cell Signaling Technology. MG-132, chloroquine and bafilomycin A1 were purchased from Sigma-Aldrich. Antibodies against SKP2 (2652), Akt (4691), p27 (3686), CUL1 (75817), CUL5 (184177) His-tag (2366) and Ubiquitin (3933) were purchased from Cell Signaling Technology. Antibodies against CHIP (55430-1-AP) and GST-tag (10000-0-AP) were purchased from Proteintech Group. Antibody against Flag-tag (F1804) was purchased from Sigma-Aldrich. Antibody against HA-tag (T501) was purchased from SAB. Antibodies against LC3B (ET1701-65), Myc-tag (R1208), GAPDH (EM1101) and β-actin (EM21002) were purchased from Hua An Biotechnology. Antibodies against SKP2 (sc-74477), CDH1 (sc-56312) and DTX3L (sc-514776) were from Santa Cruz.

**Cell culture, transfection and RNA interference**

HEK293T, A549, H1299, MDA-MB-231 and SW480 cells were cultured in DMEM containing 10% FBS (Gibco) and 100 μg/mL penicillin-streptomycin at 37 ℃ in a 5% CO_2_ incubator. Cell transfection with plasmids or siRNAs was performed using Lipofectamine 3000 (Invitrogen) according to the manufacturer's protocol. The following siRNAs were synthesized from Genepharma. The sequences of siRNAs are as follows:

si-Control: UUCUCCGAACGUGUCACGU,

si-CHIP#1: CCAGCTGGAGATGGAGAGCTA,

si-CHIP#2: CCCGAGCGCGCAGGAGCTCAA,

si-CUL5#1: CUACUGACUCUGAGAAAUA,

si-CUL5#2: GAGCAAAUAGAGUGGCUAA,

si-RNF219: GGAACUAGAAUCUCAAGUU,

si-DTX3L: GCCUUUCAACAUGCCUCAU,

si-RING1: GGCAUUGAGCUCCAGCAUU,

si-RNF2: GGCUAGAGCUUGAUAAUAA,

si-UBR5: CCAUCUACCCAAUGGCCAA,

si-AMFR: GGAAUGCACACCUUGGCUU,

si-CDH1: UGAGAAGUCUCCCAGUCAG.

**Quantitative RT-PCR**

RNA was extracted using Ultrapure RNA kit (Cwbiotech, China). cDNA synthesis and quantitative RT-PCR were performed by PrimeScript RT Master (Takara, China) and SYBR Premix Ex Taq kit (Takara, China) according to the manufacturer’s instructions, β-actin was used as internal control. The sequence of primers was listed as follows:

SKP2: forward, 5’-GCTGAAGAGCAAAGGGAGTG-3’, reverse, 5’- AGACTCATCAGACGCTAGGC-3’;

RNF219: forward, 5’-TCCTTGCAAAGAAATTATAGGAGGA-3’, reverse, 5’- TGCCCTGCACCAAGGTTAAA-3’;

RING1: forward, 5’-GGTGTCCAAGCGATCCCTAC-3’, reverse, 5’- GATCTGACCCTGGTATCGGC-3’;

RNF2: forward, 5’-ATCAGGAAGCAGGCCCTAGT-3’, reverse, 5’- TCAACAGTGGCGTTACCAGA-3’;

UBR5: forward, 5’-ACAAGCAATGCAACCAGCAT-3’, reverse, 5’- GGCGCTTTCGGTTTTCCTG-3’;

AMFR: forward, 5’-GCACCATCATCAGCGCCTAC-3’, reverse, 5’- ACTCGAAGAGGGCCAAACAC-3’;

β-actin: forward, 5’-CACTCTTCCAGCCTTCCTTC-3’, reverse, 5’-GTACAGGTCTTTGCGGATGT-3’.

**In vivo ubiquitination assay**

For SKP2 ubiquitination analysis, HEK293T cells transfected with Flag-tagged SKP2 or SW480 cells stably overexpressing Flag-SKP2 were treated with the different reagents as indicated. Cells were lysed in 1% SDS lysis buffer (1% SDS, 150 mM NaCl, 50 mM Tris-HCl [pH 7.5], 1 mM EDTA, 1 mM DTT) and boiled for 10 min. For immunoprecipitation, the lysates were diluted 10-fold in 0.25% NETN buffer (0.25% Nonidet P-40, 50 mM Tris-HCl [pH 7.5], 150 mM NaCl, 1 mM EDTA). Immunoprecipitation was performed using anti-Flag M2 beads. Analyses of ubiquitination were performed by anti-Ubiquitin blotting. As for the endogenous ubiquitination analysis of SKP2, cell lysates were immunoprecipitated with anti-SKP2 antibody (sc-74477, Santa Cruz), followed by the Western blot analyses using anti-SKP2 (2652, Cell Signaling Technology) and anti-Ubiquitin (3933, Cell Signaling Technology) antibodies.

**Co-immunoprecipitation (co-IP)**

For the co-IP assay of HSP90 and SKP2, HEK293T cells were transfected with Flag-tagged HSP90 isoforms and HA-tagged SKP2 as indicated. 24 h after transfection, cells were harvested and lysed in NP40 buffer (Beyotime) with protease inhibitors (1 mM PMSF; protease inhibitor cocktail, Merck) for 20 min on ice, and centrifuged at 13,000 g for 15 min at 4 ℃. The lysates were incubated with anti-Flag M2 beads at 4 ℃ with rocking overnight. The proteins bound to the beads were washed three times and boiled for 10 min at 95 ℃, followed by SDS-PAGE, and analyzed by immunoblotting with the indicated antibodies.

**GST pull-down assay**

The coding sequences of HSP90AA1 and HSP90AB1 were inserted into the pET-28a vector (Novagen), and the coding sequence of SKP2 was inserted into the pGEX-4T-1 vector (Amersham). GST-SKP2, His-HSP90AA1 and His-HSP90AB1 were expressed in *Escherichia coli* BL21 (DE3). To detect the direct binding, bacteria-expressed GST-tagged proteins were immobilized on GST beads (Beyotime) and then incubated with His-tagged proteins for 8 h at 4 °C under rotation. Beads were washed with GST-binding buffer (Beyotime) and proteins were eluted, followed by immunoblotting.

**Subcutaneous transplantation tumor model**

Six-week-old female BALB/c nude mice were enrolled into the study and maintained in a specific- pathogen-free environment. MDA-MB-231 cells were subcutaneously injected into the bilateral back of each mouse. The injection number of cells was 4×10^6^. The day of injection was designated day 1. After a week, mice bearing tumors were randomly assigned to control and STA-9090 treatment groups (six mice per group). Each mouse was treated with either vehicle control (20% 2-hydroxypropyl-β-cyclodextrin/water solution) or STA-9090 (5 mg/kg) via intraperitoneal injection every other day. The mice were sacrificed 5 weeks after injection. Tumor tissues were harvested, photographed, and weighed at the end of the experiment.

**Statistical analysis**

Data were analyzed using GraphPad Prism 7 software (GraphPad Software, Inc., San Diego, CA, USA). All data were presented as mean ± standard deviation. The difference between groups were assessed by Student’s *t*-test. The correlation between HSP90 and SKP2 were analyzed using Pearson’s correlation test. *P* < 0.05 was considered statistically significant, ns = not significant.


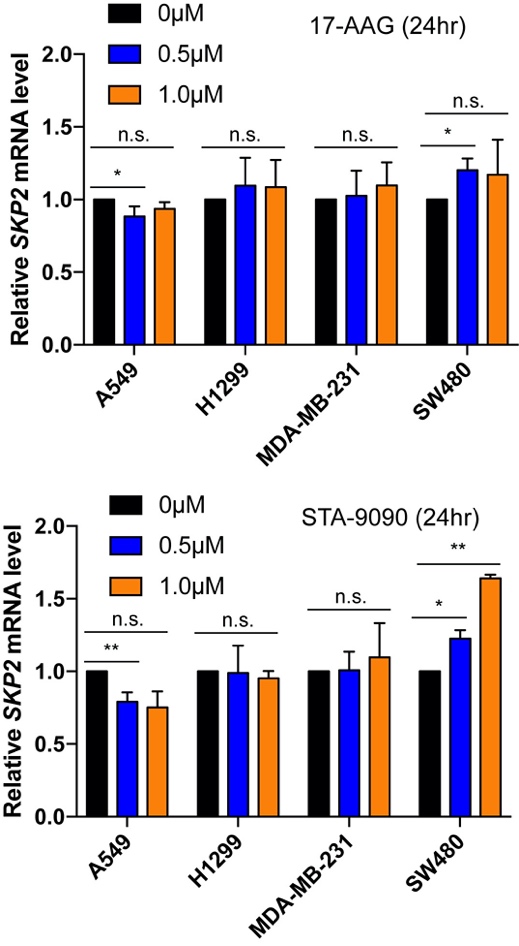


Figure. S1.

17-AAG and STA-9090 treatment had little, if any, effect on *SKP2* mRNA levels.

Transcriptional regulation of 17-AAG or STA-9090 treatment on SKP2 was analyzed by real-time PCR reaction. *, *P* < 0.05; **, *P* < 0.01; n.s., not significant.


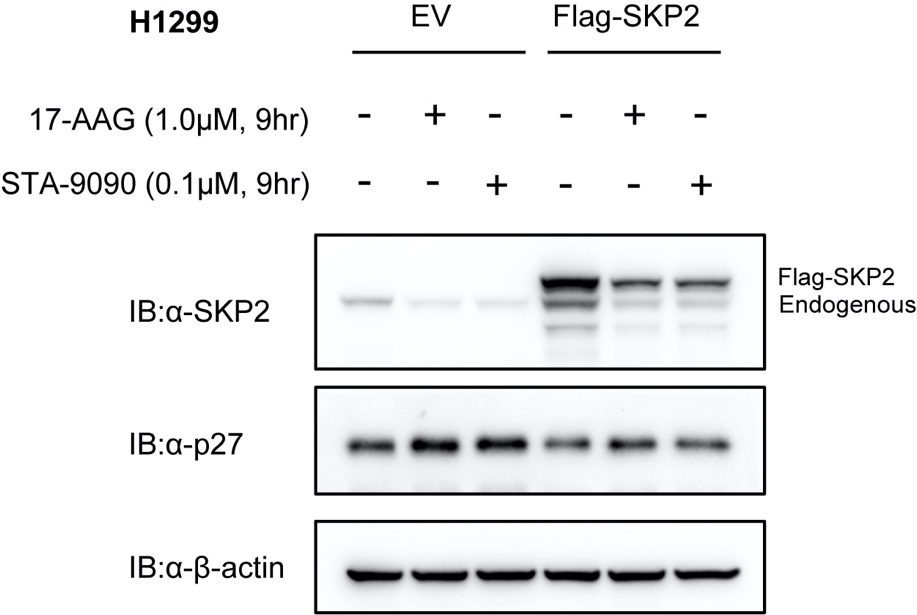


Figure. S2.

SKP2 overexpression partially rescued p27 accumulation upon treatment of 17-AAG or STA-9090.

H1299 cells were transfected with Flag-tagged SKP2 or vector control. Cells were harvested after treatment with 17-AAG or STA-9090 as indicated. The cell lysates were subjected to Western blot using indicated antibodies.


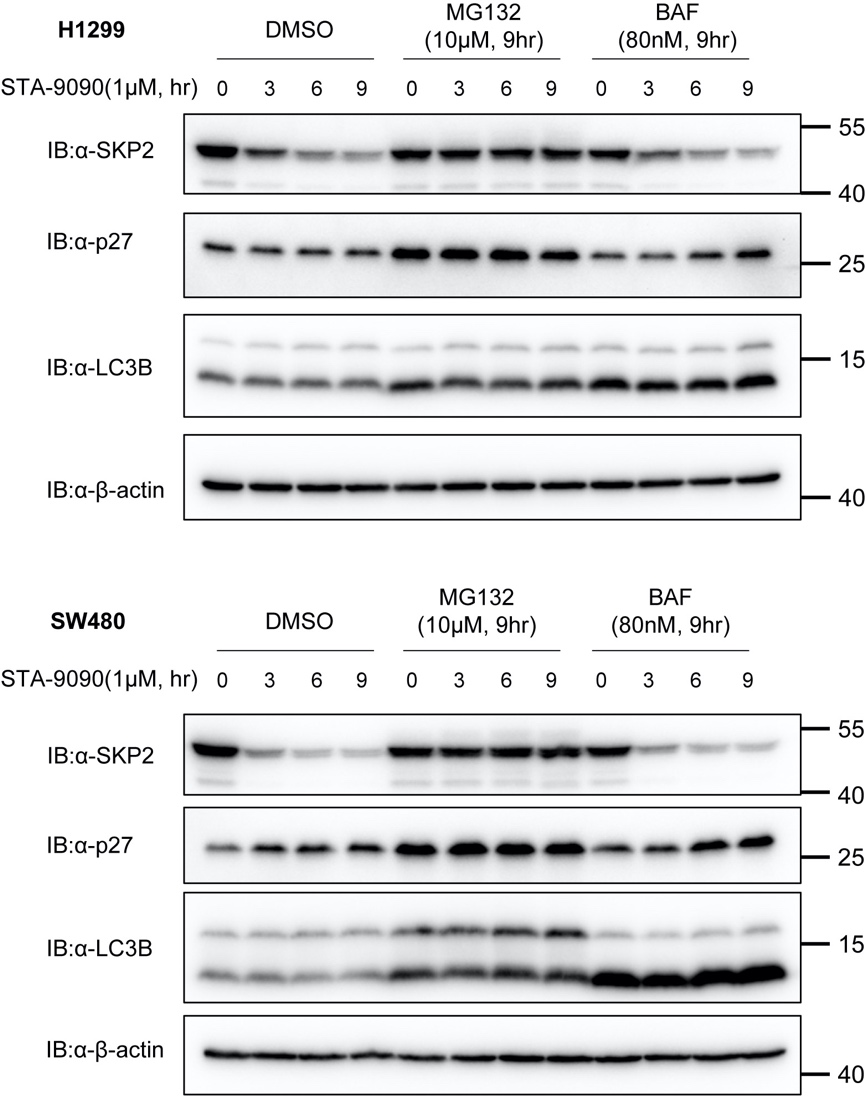


Figure. S3.

STA-9090-induced SKP2 degradation was blocked by MG132 treatment, but not by bafilomycin A1 (BAF) treatment in H1299 and SW480 cells.

H1299 or SW480 cells were lysed at the indicated time after STA-9090 treatment along with DMSO, the proteasome inhibitor MG132 (10 μM) or the lysosomal inhibitor bafilomycin A1 (BAF, 80 nM). p27 was detected as a positive control of MG132 treatment, while LC3B was used as a positive control of BAF treatment.


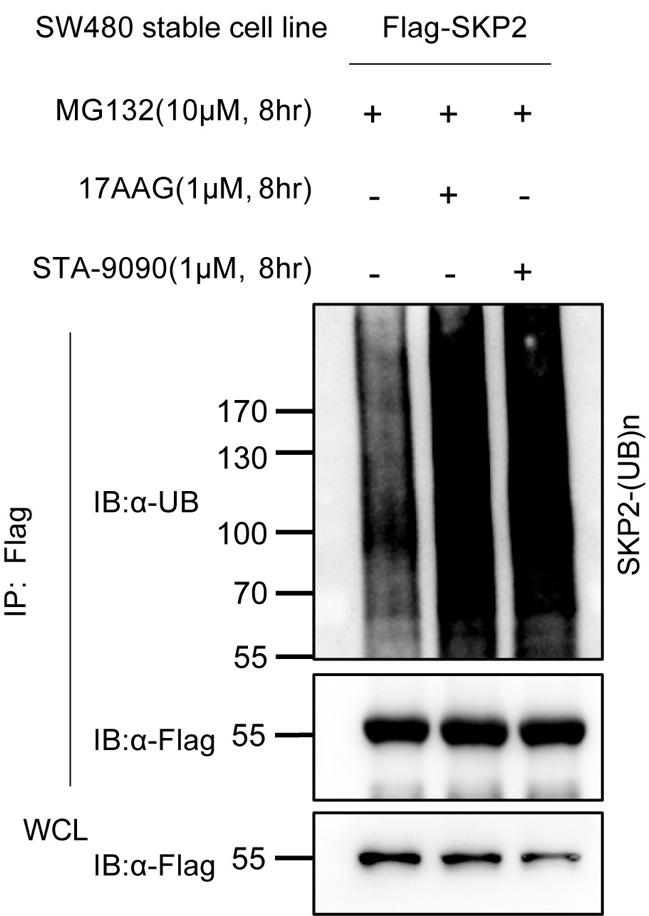


Figure. S4.

17-AAG and STA-9090 induced the poly-ubiquitination of SKP2 in SW480 cells stably overexpressing Flag-tagged SKP2.

SW480 cells stably overexpressing Flag-tagged SKP2 were treated with reagents as indicated. Cells were harvested and subjected to immunoprecipitation using anti-Flag M2 beads, followed by immunoblotting with anti-Ubiquitin antibody for the detection of poly-ubiquitination of SKP2.


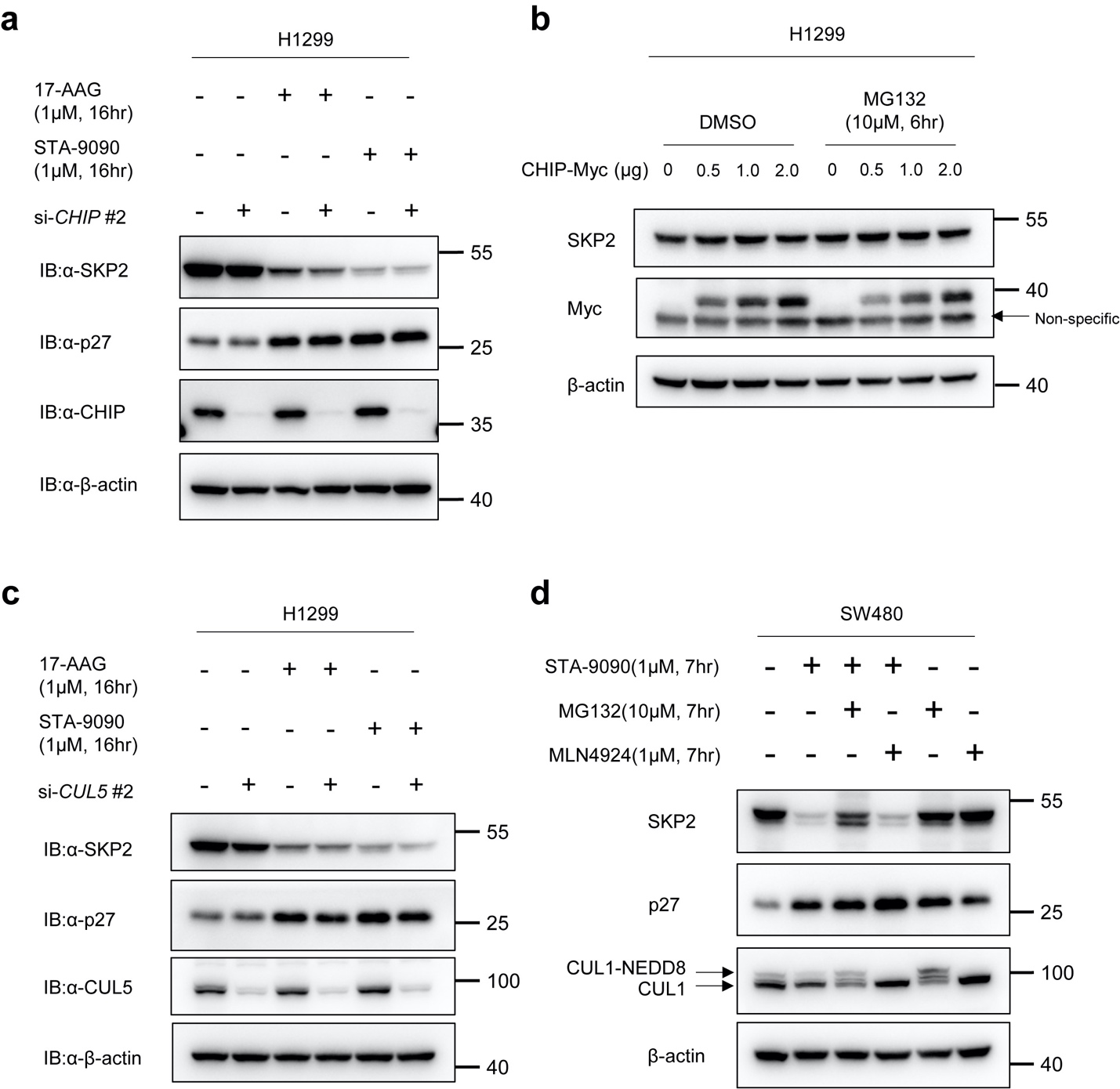


Figure. S5.

CHIP, CUL5 or CRLs are not responsible for HSP90 inhibitors-induced SKP2 degradation.

**a**, **c** CHIP or CUL5 knockdown using siRNA did not compromise HSP90 inhibitors-induced SKP2 degradation. The siRNA oligos used here were different from that of the main text (see materials and methods section for details). **b** CHIP overexpression had no effect on SKP2 degradation. **d** MLN4924 treatment did not rescue STA-9090-induced SKP2 degradation.


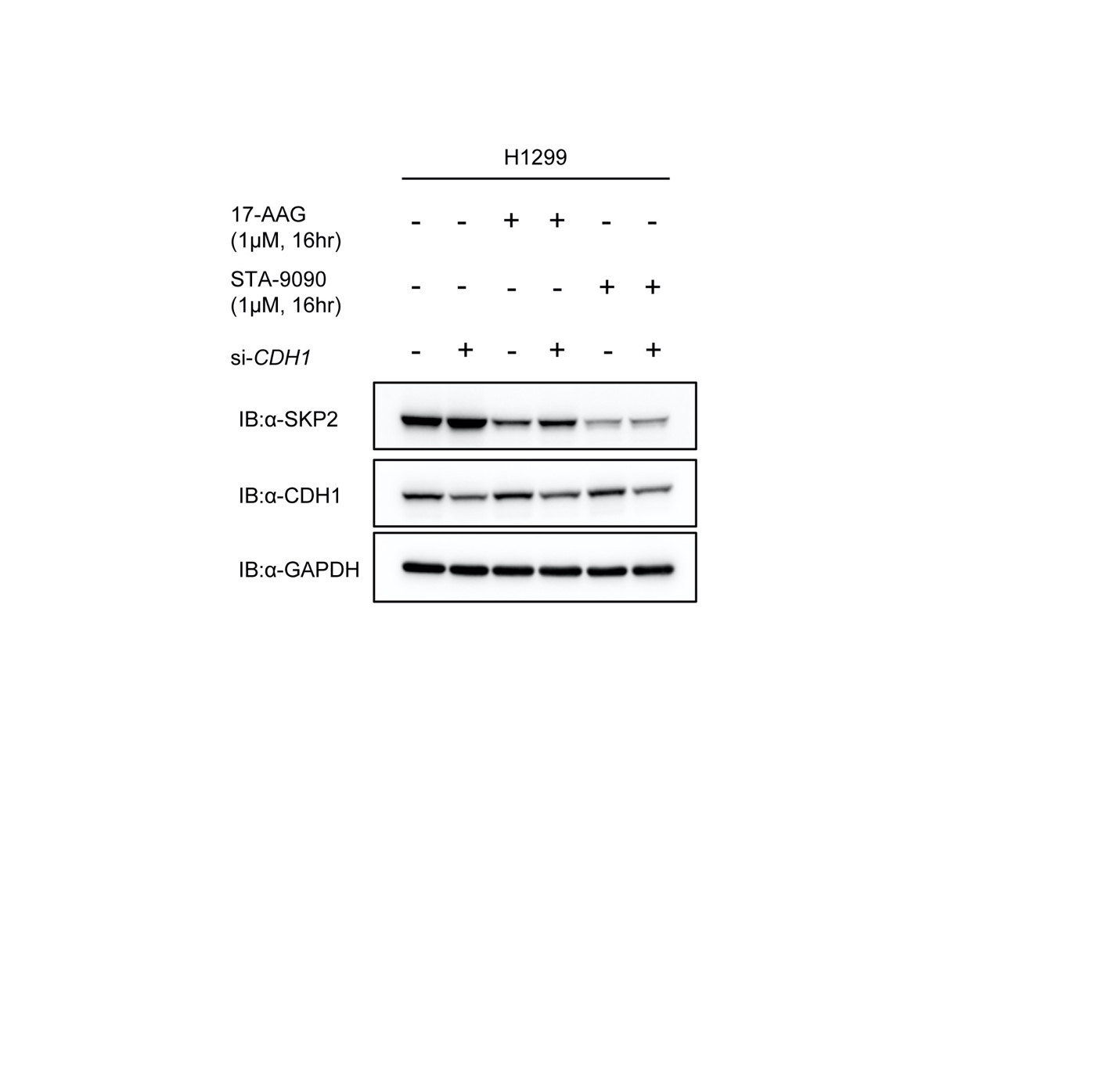


Figure. S6.

Knockdown of CDH1 only modestly recused HSP90 inhibitors-induced SKP2 degradation.


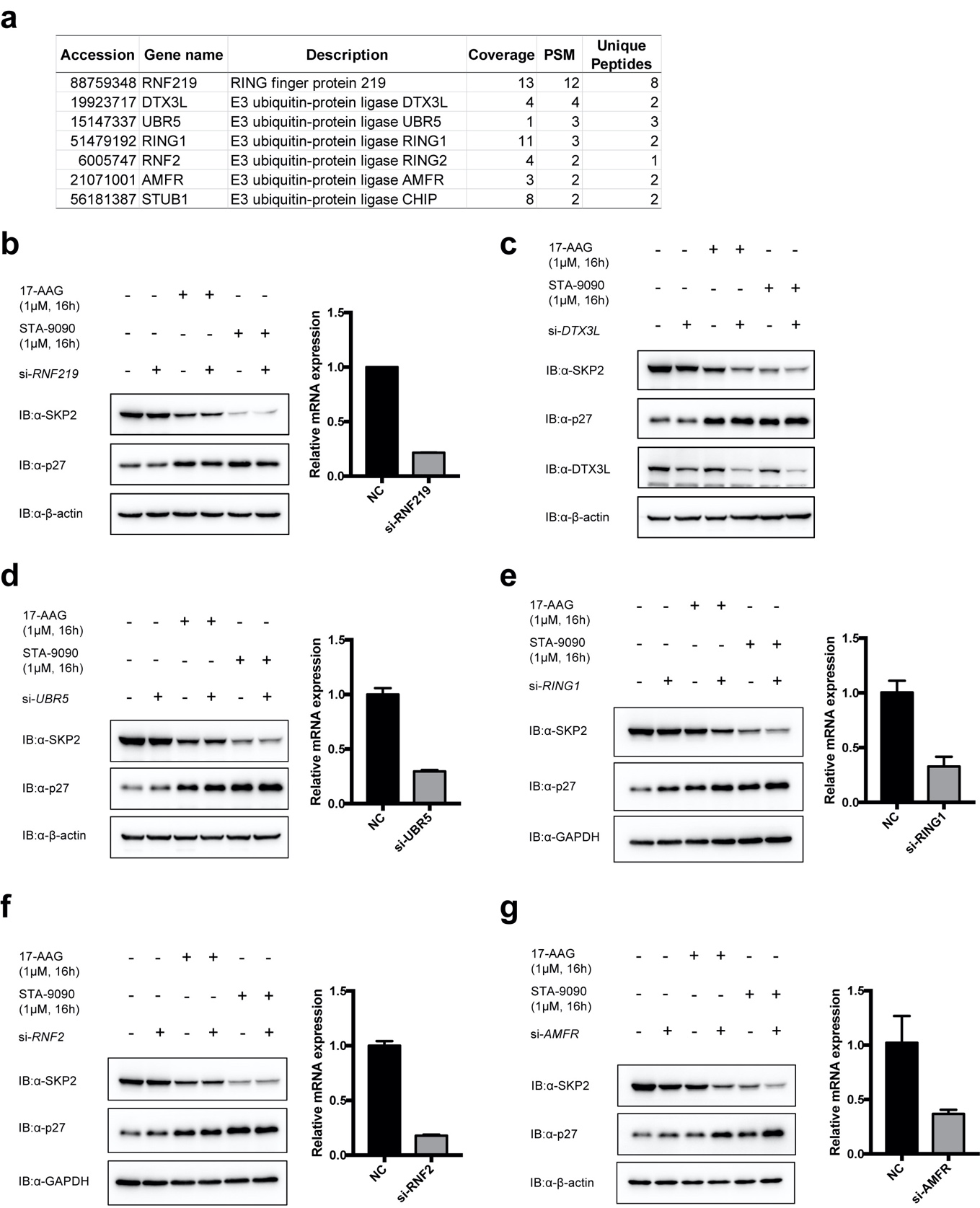


Figure. S7.

Downregulation of the putative E3 ligases did not compromise HSP90 inhibitors-induced SKP2 degradation.

**a** Identification of putative E3 ligases for SKP2 by mass spectrometry. **b-g** Knockdown of the potential E3 candidates (RNF219, DTX3L, UBR5, RING1, RNF2 and AMFR) did not rescue HSP90 inhibitors-induced SKP2 degradation.
